# Temporal Dysynchrony in brain connectivity gene expression following hypoxia

**DOI:** 10.1186/s12864-016-2638-x

**Published:** 2016-05-04

**Authors:** Brett Milash, Jingxia Gao, Tamara J. Stevenson, Jong-Hyun Son, Tiffanie Dahl, Joshua L. Bonkowsky

**Affiliations:** Bioinformatics Shared Resource, Huntsman Cancer Institute, Salt Lake City, USA; Department of Pediatrics, University of Utah, 295 Chipeta Way, 84108 Salt Lake City, UT USA; Department of Neurobiology and Anatomy, University of Utah School of Medicine, Salt Lake City, UT USA

**Keywords:** Hypoxia, Connectivity, Synapse, Axon pathfinding, Zebrafish

## Abstract

**Background:**

Despite the fundamental biological importance and clinical relevance of characterizing the effects of chronic hypoxia exposure on central nervous system (CNS) development, the changes in gene expression from hypoxia are unknown. It is not known if there are unifying principles, properties, or logic in the response of the developing CNS to hypoxic exposure. Here, we use the small vertebrate zebrafish (*Danio rerio*) to study the effects of hypoxia on connectivity gene expression across development. We perform transcriptional profiling at high temporal resolution to systematically determine and then experimentally validate the response of CNS connectivity genes to hypoxia exposure.

**Results:**

We characterized mRNA changes during development, comparing the effects of chronic hypoxia exposure at different time-points. We focused on changes in expression levels of a subset of 1270 genes selected for their roles in development of CNS connectivity, including axon pathfinding and synapse formation. We found that the majority of CNS connectivity genes were unaffected by hypoxia. However, for a small subset of genes hypoxia significantly affected their gene expression profiles. In particular, hypoxia appeared to affect both the timing and levels of expression, including altering expression of interacting gene pairs in a fashion that would potentially disrupt normal function.

**Conclusions:**

Overall, our study identifies the response of CNS connectivity genes to hypoxia exposure during development. While for most genes hypoxia did not significantly affect expression, for a subset of genes hypoxia changed both levels and timing of expression. Importantly, we identified that some genes with interacting proteins, for example receptor/ligand pairs, had dissimilar responses to hypoxia that would be expected to interfere with their function. The observed dysynchrony of gene expression could impair the development of normal CNS connectivity maps.

**Electronic supplementary material:**

The online version of this article (doi:10.1186/s12864-016-2638-x) contains supplementary material, which is available to authorized users.

## Background

Hypoxic injury complicates up to 60 % of preterm births and leads to a broad range of neurological birth defects including epilepsy, autism, ADHD, and mental retardation [1]. Despite the significant clinical impact, the specific effects of hypoxia on the developing central nervous system (CNS) are poorly characterized, and the molecular mechanisms largely unknown. Imaging studies show altered patterns of connectivity in brain magnetic resonance imaging (MRI) of children born prematurely [2, 3]. The timing of preterm birth, from 24 up to 37 weeks gestation, encompasses a key CNS developmental window characterized by widespread axon pathfinding and synaptogenesis, and thus makes the brain particularly vulnerable to disruptions of connectivity. Major pathways established during this at-risk period in premature infants include the extension of the corticospinal tracts, the development of the corpus callosum, and the formation of connections in the cortical hemispheres [4–6]. Development of connectivity in the CNS is a critical step in neural development and appears to be disrupted in disorders including autism and intellectual disability [7–9]. The development of connectivity more precisely involves the processes of axon guidance and pathfinding, synapse development and stabilization; and in the mature CNS, circuit properties and functional networks [10, 11].

Experimental studies have demonstrated that developmental hypoxia can disrupt axon pathfinding [12, 13] and alter synapse gene expression [14]. *hif1*α*,* a basic helix-loop-helix transcription factor whose protein expression is stabilized in hypoxia, controls many of the downstream responses to hypoxia including in the CNS [15]. In addition, *hif1*α is necessary for normal CNS development [16], although its precise role is unclear. A small number of target genes involved in CNS connectivity and dysregulated by hypoxia have been identified and experimentally validated; including *adenosine A2 receptor*, *Brn3b*, *EphrinB2*, *Netrin-4*, and *VAB-1* [12, 13, 17–19].

However, a global understanding of the changes in the genetic regulatory landscape of the developing CNS connectome caused by hypoxia is lacking. Developmentally, it is not known if there are critical hypoxia exposure times. Functionally, it is unclear if certain types of connectivity genes are more affected by hypoxia, and what classes or types of genes are at risk. To address these questions in a comprehensive, reproducible, and experimentally accessible fashion we performed our experiments in the small vertebrate zebrafish (*Danio rerio*). Advantages of zebrafish are that it combines the relevancy of vertebrate CNS structures and genes, with rapidity and efficiency for testing molecular mechanisms. Its low cost and ability to generate large numbers of animals in a range of experimental conditions provide significant statistical power. In addition, fundamental developmental, neurobiological, and genomic mechanisms are conserved with mammals [20–22].

Our objective was to identify the gene expression changes occurring with hypoxia in the developing CNS, and to determine if there were patterns of gene expression changes that would inform understanding of the effects of hypoxia. Our focus was on characterizing the effects of hypoxia on connectivity genes. We compared gene expression profiles across development during the first three days of zebrafish embryogenesis, when the majority of CNS development occurs, including neurogenesis, axon pathfinding, and synapse formation. Analysis was limited to those genes involved in CNS connectivity development. We found that the majority of CNS connectivity genes were unaffected by hypoxia. However, for a small subset of genes hypoxia significantly affected their expression profiles, and caused changes in both timing and levels of expression that could have significant effects on connectivity development.

## Results and Discussion

### Studying the effects of hypoxia on CNS connectivity development

**Fig. 1:**
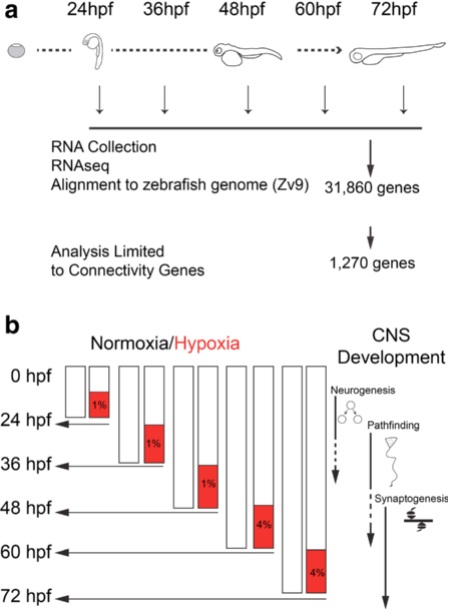
Schematic illustrations of experiments. **a** Diagram of zebrafish developmental stages (hpf, hours post-fertilization) and timing of RNA sample collection. Following collection, RNAseq, and alignment to the zebrafish reference genome, subsequent analysis was based on 1270 genes with roles in CNS connectivity development. **b** Illustration of timing of hypoxia exposure and RNA sample collection; and comparison to key events in CNS connectivity development. Percent oxygen for hypoxia shown in red boxes

To study the effects of hypoxia on development of the CNS, and in particular the effects of chronic hypoxia on the formation of connectivity in the CNS, we assayed for changes in gene expression profiles. We collected total RNA from zebrafish at five different embryonic developmental stages (24 h post-fertilization (hpf), 36 hpf, 48 hpf, 60 hpf, 72 hpf): the five stages reflect key epochs in early zebrafish development, and encompass the majority of CNS connectivity development, from neurogenesis through synapse formation (Fig. 1). For the majority of our subsequent analyses, we restricted our analysis to 1270 genes with known or likely roles in the development of CNS connectivity (Fig. 1; Additional file 1).

### Identification and cluster analysis of differentially expressed genes

**Fig. 2:**
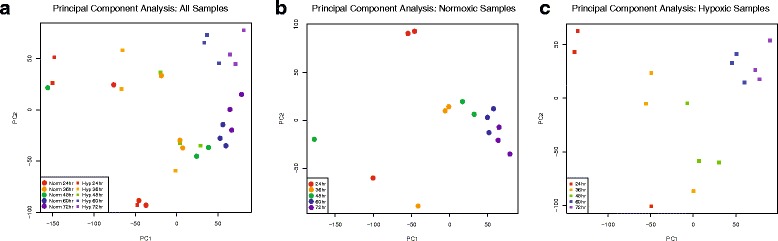
Principal Component Analysis of Gene Expression. Principal component analysis of entire data set (*n* = 31,860 transcripts) shows closer clustering at later time stages (60 and 72 hpf), but more spread at earlier time points. Separate analysis of normoxic-only or hypoxic-only data sets displays similar findings

After read mapping, gene expression was calculated using DESeq2 [23] and the abundances of genes were expressed as FPKM (fragments per kilobase of transcript per million fragments mapped). Principal component analysis (PCA) using the entire transcriptome of 31,860 genes and performed with both the normoxia and hypoxia data sets showed some separation of samples in earlier developmental stages, but tighter clustering at older ages (Fig. 2a). This trend remained when the normoxic and hypoxic samples were analyzed separately (Fig. 2b, c). The PCA results show that the RNAseq read variance for the experimental replicates at any given developmental stage or experimental condition (hypoxia or normoxia) were more similar to each other than to samples from different ages or conditions. Thus, the PCA shows that the different conditions (developmental stage; hypoxia/normoxia) were the contributors for the variance.

#### Expression analysis across development

**Fig. 3:**
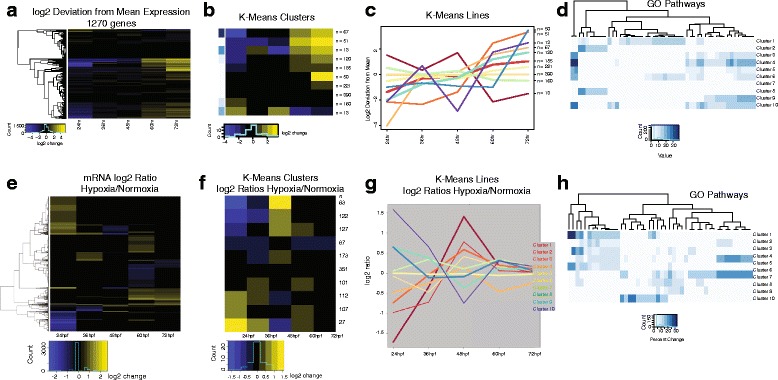
Genome-wide temporal profiles of connectivity development mRNA expression. **a** Developmental profiles of connectivity genes (*n* = 1270), displayed as a heat-map profile of groups of genes showing similar expression pattern profiles across development. **b** K-means cluster heat-map display of connectivity genes across development. **c** K clusters shown graphically as lines; relative log2 fold expression on y-axis; developmental time-points on x-axis. **d** GO pathway analysis of K clusters (see GO terms in Additional file 4: Figure S1). **e-h**, Gene expression profiles comparing hypoxia to normoxia expression profiles of connectivity genes. **e** Developmental profiles of connectivity genes (*n* = 1270), displayed as a heat-map profile. **f** K cluster heat-map display of connectivity genes. **g** K clusters shown graphically as lines; relative log2 fold expression on y-axis. **h** GO pathway analysis of K clusters (see GO terms in Additional file 4: Figure S1)

Gene expression analysis across development during normoxia showed that the 1270 genes fell into ten K-means clusters (Fig. 3a-d). Differentially expressed genes, compared to their average expression across development, were defined as genes with a false discovery rate (FDR) less than 0.1 and a more than a 2-fold expression change (Additional file 2).

#### Expression analysis comparing hypoxia and normoxia and across development

**Fig. 4:**
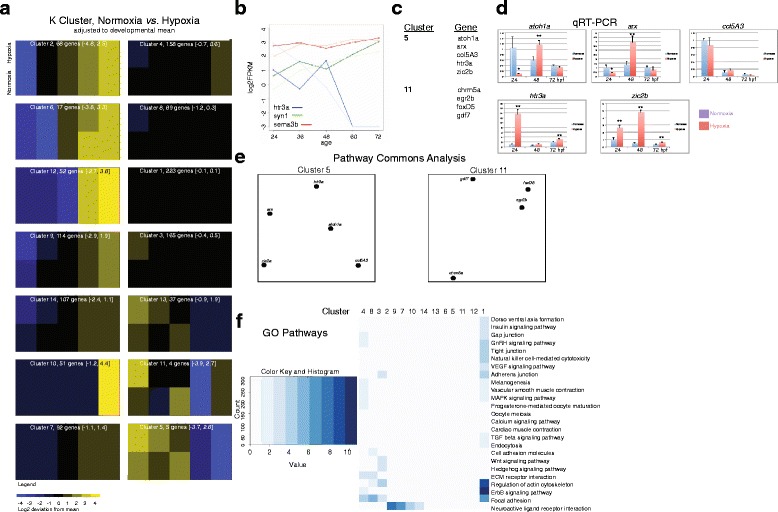
Normalized K cluster analysis demonstrates altered timing of expression caused by hypoxia. **a** K cluster groups shown as heat maps, in which gene expression is averaged across development, and deviations both from the developmental average, and hypoxia versus normoxia, are shown. Values in parentheses indicate the range of log2 fold change of genes in the cluster. **b** Expression analysis of three representative gene expression changes from hypoxia across development shown as lines; lighter shade indicates expression in hypoxia. X-axis developmental age, y-axis log_2_FPKM. **c** Genes in clusters 5 and 11; transcription factors are disproportionately represented. **d** qRT-PCR of genes from cluster 5; error bars standard deviation; two-tailed *t* test; * *p* < 0.05; ** *p* < 0.01. **e** Pathway Commons Network analysis, displayed with PCViz, shows no shared paths between the genes in cluster 5 or 13. **f** KEGG analysis of K clusters. GO categories are only loosely organized into clusters, indicating that the effects of hypoxia on gene expression are not based on gene type/category

For hypoxia, the relative expression of the genes was compared to their normoxic levels (Fig. 3e-h; Additional file 3; Additional file 4). To adjust for changes in levels due to changes associated with development, we normalized expression for each gene to its mean level across development in each experimental condition (normoxic or hypoxic), and then compared hypoxic to normoxic levels at each developmental time-point (Fig. 4a; Additional file 5). We found that the 1270 genes grouped into fourteen K-means clusters. The largest cluster (223 genes), cluster 1, had minimal changes across development or with hypoxic conditions. Several other clusters showed a developmental increase in gene expression but with little or no effect from hypoxia (clusters 2, 7, 10, 12; total of 526 genes). In total 749 genes (59 %) were unaffected by hypoxia. The remaining clusters demonstrated differential responses of gene expression to hypoxia during development. Expression analysis showing three representative genes from clusters 5, 8, and 14 displayed as lines demonstrates how different genes had differential responses to hypoxia across development (Fig. 4b). Interestingly, Clusters 5 and 11, which displayed the most dynamic responses to hypoxia, contained a total of only nine genes. However, although transcription factors constituted a majority of the two clusters (Fig. 4c, d; Additional file 6), there were not other apparent shared biological features of the clusters, including a Pathway Commons Network analysis showing no direct protein-protein interactions between the members of clusters 5 or 11 (Fig. 4e) [24]. GO pathway analysis was most descriptive for cluster 1, the largest cluster; while clusters 2, 7, and 10, which showed increases in expression during development but no effect from hypoxia, contained a higher proportion of receptor/ligand genes (Fig. 4f). Thus, there did not appear to be a certain type or category of genetic response to hypoxia, as least as tested with Pathway Commons Network analysis or GO pathway analysis.

**Fig. 5:**
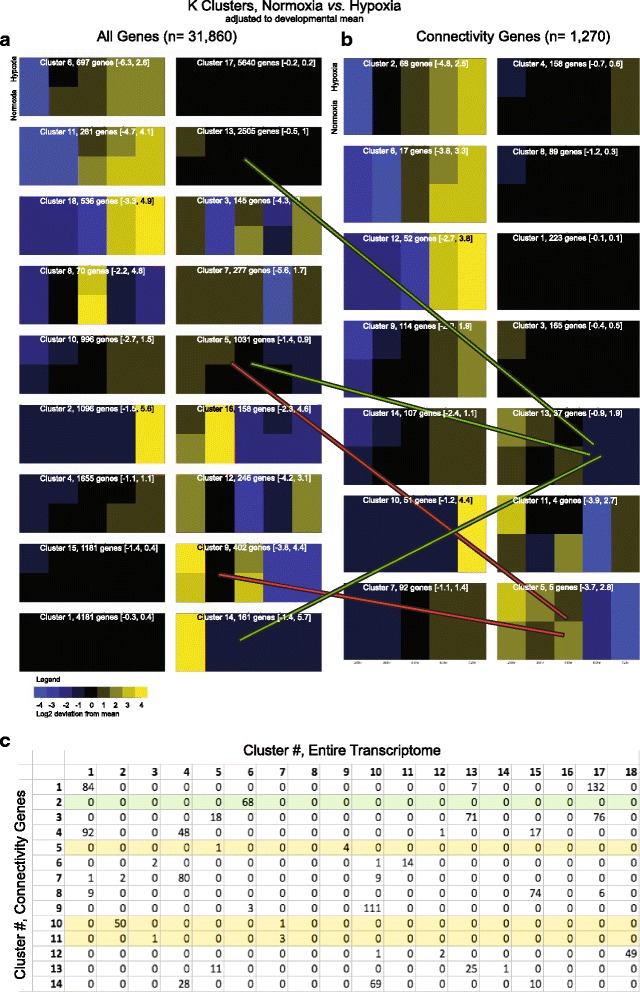
Normalized K cluster analysis of all genes compared to connectivity genes only shows improved resolution of expression differences. **a** Analysis of all genes (*n* = 31,860), K cluster groups shown as heat maps, in which gene expression is averaged across development, and deviations both from the developmental average, and hypoxia versus normoxia, are shown. Values in parentheses indicate log2 range fold change of genes in the cluster. **b** Examples of differential assignment of genes to different clusters (heat-maps of connectivity genes is from Fig. 4a). Green lines show different groups of genes assigned to different clusters in the two analyses for cluster 13. Red lines show differential assignment for cluster 5. **c** Table representation of differences in gene assignment to K clusters, comparing all genes (columns) to connectivity genes only (rows)

We also tested whether analysis of the entire original transcriptome (*n* = 31,860) would result in an alternative pattern of clustering of expression changes. K-means analysis of the entire transcriptome resulted in eighteen clusters. Genes that clustered together in the connectivity gene analysis were often split into different clusters when the entire transcriptome was analyzed (Fig. 5; Additional file 7). The K cluster analysis for the entire transcriptome often provided decreased resolution compared to when only connectivity genes were analyzed. For example, some genes from clusters 5 and 9 in the pan-transcriptome analysis, when analyzed in the connectivity gene set could be observed to in fact have differential expression profiles at 24 and 48 hpf (Fig. 5b, red lines). However, it is reassuring that for most genes their cluster assignments and grouping remained constant in both the whole transcriptome and connectivity-only analyses (Fig. 5c). Of note, analysis of the entire transcriptome included the *hypoxia-inducible factor* (*hif*) genes. *hif1ab*, *hif1an,* and *hif2*(*epas1a*) were in cluster 1; *hif2(epas1b)* and *hif1al2* were in cluster 13; and *hif1aa* was in cluster 14. Thus, we think that the use of the entire transcriptome for K means clustering does not provide additional insight and actually obscures some of the findings from analysis of the 1270 connectivity genes.

#### in vivo testing, qRT-PCR validation, and biological significance

**Fig. 6:**
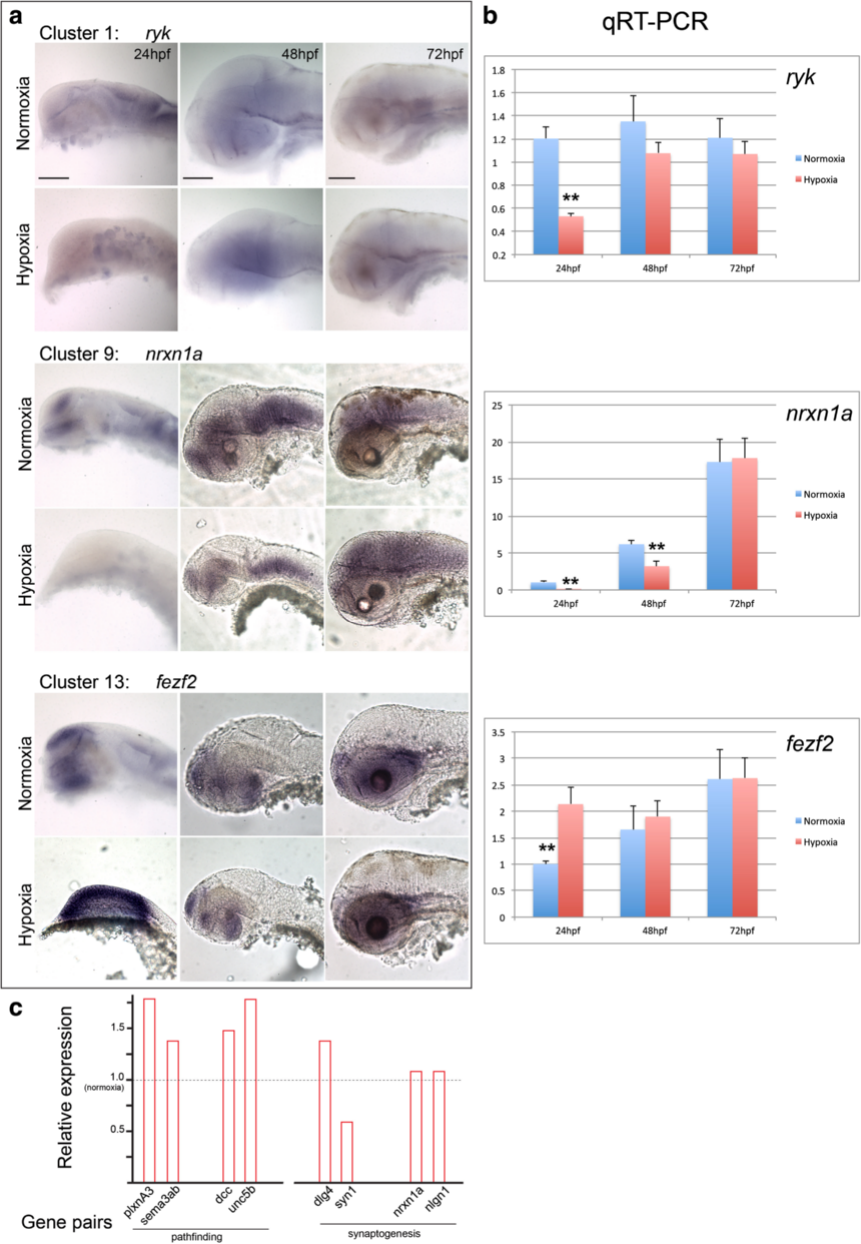
in situ validation of RNAseq results, and schematic of hypoxia-associated dysynchrony. **a** Examples of gene expression changes across development, and hypoxia compared to normoxia. Clusters refer to K analysis, Fig. 4. Whole-mount in situ images for *ryk*, *nrxn1a*, and *fezf2*; lateral views, dorsal to top, rostral to left. Scale bar 50 μm. *ryk* expression is decreased in hypoxia at 24 hpf, but then is otherwise relatively invariant across development and in hypoxia compared to normoxia. *nrxn1a* and *fezf2* also demonstrate dynamic changes in expression at different developmental stages, and in hypoxia/normoxia. **b** qRT-PCR results for *ryk*, *nrxn1a*, and *fezf2;* normalized to *elfa* with relative value set to “1” for 24 hpf normoxia. Error bars, standard deviation; two-way *t* test; ** *p* < 0.01. **c** Schematic of effects of hypoxia on disrupting normal connectivity gene expression interactions. Relative expression at 24 hpf is shown for axon pathfinding and at 72hpf for synaptogenesis. Normal/normoxic expression level is set at “1”; fold-change of gene expression following hypoxia is shown by the red bars. Two examples each of ligand/receptor gene pairs are shown for axon guidance; and two pairs of pre-/post-synaptic genes for synaptogenesis

To perform testing of the RNAseq results and to validate RNAseq results for individual genes, we performed experiments in the embryonic zebrafish and compared hypoxic to normoxic animals. We analyzed expression for three genes from three different clusters by both in situ hybridization and quantitative RT-PCR (Fig. 6a, b; Additional file 8). *ryk*, a receptor-tyrosine kinase from cluster 1, has decreased expression at 24 hpf in hypoxia (fold change 0.33; two-way *t* test *p* < 0.001), but otherwise is relatively constant across development and in normoxia versus hypoxia. *nrxn1a*, a synaptic transmembrane protein in cluster 9, demonstrates increased expression levels at later developmental stages. However, hypoxia diminishes expression at 24 hpf and 36 hpf (fold changes 0.13 and 0.52; two-way t tests *p* < 0.01). *fezf2*, a transcription factor important in neuronal fate specification in cluster 13, had higher levels of expression in early development. Interestingly hypoxia caused a significant increase in expression levels at 24 hpf (fold change 2.1; two-way *t* test *p* < 0.01). Thus, for these genes the qRT-PCR results match the in situ results. However, there are a few minor discordances compared to the RNAseq results (Additional file 5); for example for *ryk* at 24 hpf, *nrxn1a* at 48 hpf, and *fezf2* at 72 hpf, the RNAseq change in hypoxia is discordant with the in situ and qRT-PCR results, emphasizing the importance of follow-up experimental validation.

The temporal changes demonstrated that hypoxia could affect gene regulation and action by changes in overall expression levels. We then considered whether hypoxia could cause a dysynchrony by altering relative expression of genes known to interact, for example receptor-ligand pairs. A change in relative expression of one or both genes that interact could potentially have additive effects on disrupting signaling. We examined four pairs of genes with potential interactions and roles in connectivity: *plxnA3* and *sema3ab*; *dcc* and *unc5b*; *dlg4* and *syn1*; and *nrxn1a* and *nlgn1* (Fig. 6c). At 24 hpf *plxnA3* and *sema3ab* were both up-regulated by hypoxia; these genes are a cell-surface receptor/ligand pair [25], and increased expression of both genes would disrupt normal axon guidance. *dcc* and *unc5b* at 24hpf are also up-regulated by hypoxia; both genes are receptors for *netrin-1* and are necessary for normal midline axon guidance [26]. Examination of the effects of hypoxia at 72 hpf showed minimal effects on *nrxn1a* and *nlgn1*, which have roles in synapse adhesion [27]. *dlg4*, a post-synaptic scaffolding protein, and *syn1*, a pre-synaptic vesicle-associated protein, show contrasting up- and down-regulation by hypoxia at 72 hpf, which could interfere with normal synaptic function.

**Fig. 7:**
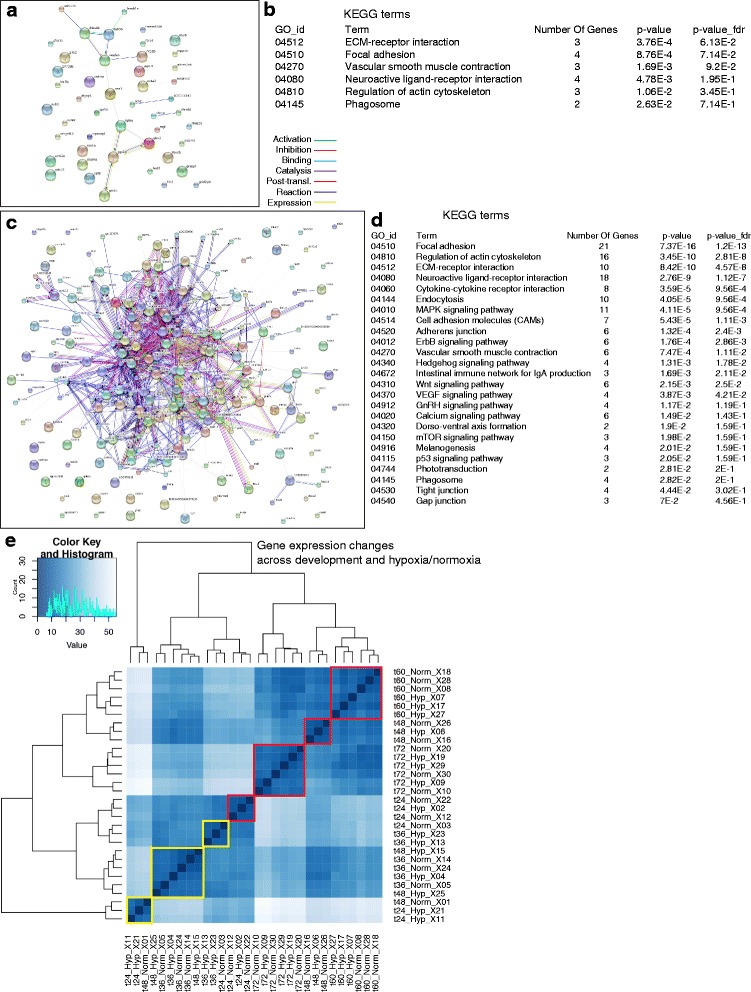
Protein-Protein Interactions Network. **a** STRING analysis of most significant (adjusted *p* < 0.05) genes interactions, *n* = 57; color key for interaction type is shown to the right (Additional file 10: Figure S2 shows enlarged picture and fonts). **b** KEGG analysis of the most common pathways for pathways with 2 or more genes. **c** STRING analysis with relaxed criteria (unadjusted *p* < 0.05), *n* = 244 (Additional file 10: Figure S2 shows enlarged picture and fonts). **d** KEGG analysis of pathways using relaxed criteria for pathways with 2 or more genes. **e** Heat-map profile of gene expression changes across development and experimental conditions. Red boxes show clusters in which all the groups had the same age but had both hypoxic and normoxic samples. Yellow boxes show clusters in which both the age and the experimental condition varied. At younger ages hypoxia is noted to have a larger effect by causing differential clustering based on the presence of hypoxia/normoxia (yellow boxes) rather than based solely on age (the red boxes)

To test whether the connectivity genes fit into known protein interaction networks we analyzed genes with the most significant changes from hypoxia, while controlling for their expression changes across development (Fig. 7; Additional file 9). The differential expression test was applied to each gene individually, and a *p* value was determined for each individual gene. Then an adjusted *p* value was calculated from all the individual *p* values with adjustment for multiple testing. 57 genes had adjusted *p* values < 0.05, but only a minority of this group (~15) had direct protein-protein interactions with each other (Fig. 7a) [28], and KEGG term analysis had sparse representation in a few groups (Fig. 7b), and GO term analysis revealed no statistically significant pathway memberships. 244 genes had unadjusted *p* values < 0.05 (no adjustment made for the multiple comparisons), and showed multiple interactions in many KEGG pathways (Fig. 7c, d). An enlarged figure (Additional file 10: Figure S2) shows gene names more clearly for Fig. 7a, c.

When we compared overall gene expression changes with development and with experimental condition, we found that at later ages genes clustered primarily by developmental time, and less by hypoxia versus normoxia (Fig. 7e). In contrast, at earlier ages the relative effects of hypoxia were more significant, and expression changes clustered in a mixture of time and hypoxia effects. With this analysis the dysynchrony of expression is more notable- for example, that the gene expression profile of animals at 36 hpf of normoxia is more similar to that of hypoxic animals at 48 hpf.

## Conclusions

We combined a high-resolution transcriptional time course analysis, developmental comparisons of expression, and effects of hypoxia, to characterize and validate the role of hypoxia on connectivity gene expression in the CNS. We had several major findings. First, we found that the majority of CNS connectivity genes are not significantly affected by hypoxia during development. Second, for the minority of connectivity genes which are affected by hypoxia do not represent a single type or category of gene or protein. Third, connectivity genes affected by hypoxia display a dysynchrony. That is, their relative expression level is aberrantly increased or decreased at an inappropriate time of development. Hypoxia also altered expression levels at defined developmental times of interacting gene pairs, including axon guidance ligand/receptor pairs and synaptic proteins, that would be predicted to disrupt their normal combinatorial functioning. While following hypoxia embryos showed a slowing in their developmental progression, the gene response to hypoxia was not simply a delay in the timing of their normal expression, and in some cases the hypoxia in fact caused an “acceleration” with increased levels of expression.

Previous work from our group and others has shown that hypoxic injury can disrupt the normal development of axon and synaptic connectivity [12–14, 29]. The work presented here extends those findings by demonstrating that hypoxia acts at least in part by a causing a dysynchronous response of genes including of interacting receptor/ligand pairs. For example, the receptor/ligand pair *plxnA3* and *sema3ab* are both up-regulated by hypoxia, which could lead to elevated GTPase activity and increase repulsive axon guidance [30]. Or, as we found, hypoxia causing a decrease in pre-synaptic *syn1* coupled with a post-synaptic increase in *dlg4*, could alter synaptic stabilization and function [31, 32].

While the changes in gene expression reveal some of the functional logic of the hypoxic response in our analysis we are not able to differentiate direct or indirect effects. Genes could be directly regulated by hypoxia-responsive transcription factors; or could be downstream targets in a multi-step cascade. Our findings provide an excellent starting point for deciphering the regulation of genes by hypoxia. In addition, future experiments can address why certain genes are particularly vulnerable to hypoxia, which has important implications for understanding the effects of hypoxia on CNS connectivity development. In our analysis we focused our attention on a subset of 1270 genes with known roles in connectivity development, in particular on genes with roles in axon pathfinding and synapse development and stabilization. Since the genes regulating connectivity development are relatively well-established [33], our limitation of analysis to this subset provided an enhanced ability to detect trends and patterns that would otherwise have been obscured by including the entire transcriptome.

Future studies could also examine whether certain neuron groups are more susceptible to the effects of hypoxia. While we used RNA from the head and brain, changes in expression from other non-neural tissues could have affected our results. Subsequent studies could use genetically-defined methods to limit analysis to neural tissue only, for example with fluorescence-activated cell sorting (FACS) analysis or translating ribosomal affinity purification (TRAP) [34].

The transcription factor *hif1*α is a master regulator of the cellular response to hypoxia [15] along with a collection of other *hif* genes and transcription factors [35]. *hif1*α is necessary for normal brain development [36], but in the developing brain hypoxia or ectopic expression of *hif1*α interrupts normal axon guidance [12]. This adverse effect of hypoxia on connectivity has also been observed to affect synapse development in a rodent model [14]. When we analyzed the changes in expression of the different *hif* isoforms in response to hypoxia we noted relatively minor changes. This is similar to a previous report in zebrafish [37], and is likely due to the regulation of *hif* by hypoxia predominantly occurring at the post-transcriptional (protein) stage [38].

Chronic hypoxia and injury to the developing brain in premature infants can lead to adverse neurocognitive and neurodevelopmental outcomes [39]. Premature infants can experience extended bouts of hypoxia [40, 41], and MRI studies have demonstrated altered connectivity in ex-premature infants [42, 43]. Our findings suggest that certain key genetic pathways may be affected in premature infants by hypoxia.

In summary, our data suggests two central findings concerning the effects of hypoxia on CNS connectivity development. First, that the major effects of hypoxia are due to a dysynchrony of gene expression; and second, that hypoxia disproportionately affects a subset of connectivity genes. These results should lead to further investigations on why certain genes are prone to the effects of hypoxia, and what effects those gene responses have on the developing CNS.

## Methods

### Ethics statement

Zebrafish experiments were approved and performed under guidelines from the University of Utah Institutional Animal Care and Use Committee (IACUC), and regulated under federal law (the Animal Welfare Act and Public Health Services Regulation Act) by the U.S. Department of Agriculture (USDA) and the Office of Laboratory Animal Welfare at the NIH, and accredited by the Association for Assessment and Accreditation of Laboratory Care International (AAALAC).

### Fish stocks and embryo raising

Adult fish were bred according to standard methods [44]. Strain AB was used for all experiments. Embryos were raised at 28.5 °C in E3 embryo medium. For in situ staining embryos were fixed in 4 % paraformaldehyde (PFA) in PBS overnight (O/N) at 4 °C, washed briefly in PBS with 0.1 % Tween-20, dehydrated, and stored in 100 % MeOH at −20 °C until use.

### Hypoxia

Embryonic zebrafish were placed in a sealed plexiglass chamber connected via a controller that monitored and adjusted nitrogen gas flow to a desired pO_2_ set point (Biospherix Ltd.) (full details in [12]). All solutions were pre-equilibrated to either normoxia or hypoxia for at least 4 h before use, and embryos were transferred into and out of pre-equilibrated solutions. Following hypoxia exposure, embryos were returned to media kept in normoxic conditions. Timed staging was used to determine age at fixation for analyses and RNA collection.

### RNA extraction, cDNA preparation, and sequencing

Embryo heads were manually prepared by dissection with a tungsten needle. Following dissection, embryo heads were triturated in Trizol with a 25-gauge needle on a 1 mL syringe. RNA was prepared with a miRNeasy kit (Qiagen). Quality of total RNAs was checked using a NanoDrop 2000 Spectrometer and an Agilent 2100 Bioanalyzer. RNA integrity number (RIN) from the Bioanalyzer ranged from 7.2 to 9.7 (average 9.1; median 9.4). Library preparation and sequencing was done at the University of Utah Microarray and Genomic Analysis Shared Resource on a Hiseq 2000 machine and processed using the Cassava 1.8 pipeline. We generated 50 base single-end reads from the RNA libraries. 30 samples were multiplexed across 5 lanes of a single flow cell, for a total of 6 samples per lane. We generated on average 25,073,213 reads per biological replicate. Raw sequence data has been uploaded to the Sequence Read Archive under BioProject PRJNA293106 (http://www.ncbi.nlm.nih.gov/bioproject/PRJNA293106) where the per-sample sequencing yield is available.

Total RNA from zebrafish was collected at 24hpf, 36hpf, 48hpf, 60hpf, and 72hpf; with biological triplicates for each stage (time-point) and for each experimental condition. For RNAseq and qRT-PCR, pools of ~30 embryos for each sample (stage and experimental condition) were collected.

### RNAseq alignment

Raw sequence data quality was checked using FastQC version 0.10.1 (http://www.bioinformatics.babraham.ac.uk/projects/fastqc/). No sequence quality issues were found so no adapter or quality trimming was performed prior to genome alignment. We did perform adapter trimming of the RNA reads during alignment to the zebrafish Zv9 genome and known splice sites using the novoalign aligner (www.novocraft.com). Mapping was done using Novoalign version 2.0.8 (Novocraft Inc, http://www.novocraft.com) with default parameters except output was set to SAM format, and one random alignment was chosen for fragments aligning to multiple locations (−r Random). Reads were aligned to the Zv9 zebrafish build [20] with splice junctions derived from the UCSC Ensembl refFlat gene table (http://genome.ucsc.edu/goldenpath/gbdDescriptionsOld.html#RefFlat).

### RNA preparation in normoxia and hypoxia during development

For hypoxia, we placed developing embryos in pre-conditioned media in a sealed plexiglass chamber set to a reduced O_2_ concentration [12] for 12 h periods. Immediately following hypoxia (or normoxia) embryos were sacrificed and RNA collected. Based on our previous studies of the role of hypoxia on CNS development and effects on lethality [12], we used 1 % pO_2_ for ages up to 48 hpf (hours post-fertilization); and 4 % pO_2_ for older ages. At the time-point for collection embryos were dissected and only head tissue was used for subsequent RNA preparation.

### Transcript/genomic alignment

Following sequencing, RNA reads were aligned to the zebrafish genome and splice junctions with genome build Zv9 [20]. A total of 31,860 different transcripts, obtained from the UCSC Ensembl RefFlat table, were tested for differential expression using DESeq2.

For the majority of our subsequent analyses, we restricted our analysis to 1270 genes with known or likely roles in the development of CNS connectivity. The 1270 genes were identified based on the gene ontology (GO) terms "axon guidance" or "synapse". The search for genes meeting the GO criteria was performed separately in the zebrafish and human genome assemblies; the lists of genes were merged and the zebrafish orthologs were then substituted for any genes only present on the list from the human genome due to incomplete annotation of the zebrafish genome. The file was then manually reviewed for omissions, and the following genes with known (published) roles in CNS connectivity development were manually added: *apoeb, appb, ctbp2, dlg4, ephrinB2a, foxP2, fzd3a, lamb1a, lamc1, mnx1, nav1, neo1, sema3b, sparc, sparcl1, tgfbr2, thbs1, thbs2*, and *thbs4*.

### K cluster and statistical analyses

Gene expression analysis was performed using the Useq 7.8.1 software package with default parameters [45]. Differentially expressed genes were defined as genes with a false discovery rate less than 0.1 and more than a 2-fold expression change.

K-means clustering on log_2_-transformed FPKM values was performed using R version 3.1. The value of K was chosen by plotting the total within-cluster variance as a function of K, identifying the minimum K for which the total within-cluster variance was less than 20 % of the total variance and the variance curve showed a marked decrease in slope as K increased. For normoxic samples K = 10 met these criteria. For samples with normoxic and hypoxic data sets, K = 14 was selected.

### Pathway analysis

KEGG and GO enrichment was analyzed using DAVID (http://david.abcc.ncifcrf.gov) [46, 47] functional annotation tools with default parameters. Each cluster of genes identified by K-means clustering was annotated at the DAVID site. The KEGG pathway/gene cluster associations were hierarchically clustered using the –log_10_(p) value of the pathway enrichment. Clustering was performed in R version 3.1 using gplots (http://cran.r-project.org/web/packages/gplots/index.html).

Pathway Commons Analysis was performed using PCViz (http://www.pathwaycommons.org/pcviz/) [24].

### Principal Component Analysis

The principal components analysis was performed in R (version 3.1.2) using the prcomp function (https://stat.ethz.ch/R-manual/R-patched/library/stats/html/prcomp.html). Log-scale FPKM values were centered and scaled in the prcomp function prior to analysis.

### in situ *hybridization*

Whole-mount in situ labeling for *ryk, neurexin1a,* and *fezf2* was performed as previously described [48]. Briefly, embryos were fixed overnight in 4 % paraformaldehyde/PBS at 4 °C and stored in 100 % methanol. Embryos were permeablilized with 10 μg/ml proteinase K, and then digoxigenin-labeled probes were hybridized and detected with alkaline phosphatase-conjugated anti-digoxigenin Fab fragment (1:5000, Roche) and BM purple alkaline phosphatase substrate. 30 or more embryos were analyzed for each probe and condition. Probes were generated by PCR amplification of genomic exon coding sequence and cloned into pCR4-TOPO (Invitrogen). Primers were (forward; reverse; listed 5’ to 3’): *ryk*, CGCATACGGTACAGTGGAGA; TTGACCTCTTCCTCGCTCAT; *neurexin1a*, CTGCGAAAGCGAAATGAGTT; GACTCCTCCATTCAGGCAAA; and *fezf2*, GCAAGCCAAGGCTTTAATGA; TTCCCGTCTGAAGAGCAGTT.

### Microscopy and image analysis

Following in situ staining, embryos were transferred step-wise into 80 % glycerol/20 % PBST, mounted on a glass slide with a #0 coverslip placed over a well made using electrical tape, and imaged on a compound light microscope. Illustrations were composed with Adobe Photoshop and Illustrator.

### Quantitative RT-PCR

New samples were prepared separately for the RT-PCR with three independent biological replicates. Real-time PCR was performed on an ABI PRISM 7900HT (Applied Biosystems) with SYBR Green fluorescence label. qPCR for *ryk, nrnx1a, fezf2, atoh1a, arxa, col5A3a, htr3a and zic2b* was performed on cDNA prepared from 24 hpf, 48 hpf and 72 hpf embryos. Each reaction was performed in triplicate and the mean of replicates was calculated; results were normalized to the mRNA level of each gene in normoxic embryos at 24 hpf with *elongation fator I alpha* (*elfa*) transcript levels as a control. Primers were as follows (shown as 5’ to 3’, forward; reverse): *ryk*, ACACCTGTCACAAGTTATCCAT, AGACGTCTCGAAGTGTGACT; *nrxn1a*, CCACAACTCCACAGGACGAT, GGGAAGTGGTGGGTGAGCT; *fezf2*, GGCAACTGGACCAAATCGTG, AGTGCGCGTTGAAGACCTTT; *atoh1a*, GAGAGTTCTCGCCTCACTCG, TCCGGCGGTGTGTTTTCTTA; *arxa*, GACAACCGAAGTCACCTCCAA, TCTAGGTGCTCGTGAAACCC; *col5A3a*, TCAGGACAGCAGTCCCTCTAC, TTAATCGGCCTCTCCTGCTTTG; *htr3a*, TGGAGTTCAGCCTGCATCAC, ACTGAGCAATCCACCACTGT; *zic2b*, AACACATGAAGGTTCACGAGGA, AGAATCAGGCGACAAGGTGC; *elfa*, CTTCTCAGGCTGACTGTGC, CCGCTAGCATTACCCTCC. Reactions were run with the following conditions: 95 °C for 10 min and 40 cycles of 95 °C 20 s/60 °C 20 s/72 °C 40 s. The relative levels of each target mRNA were normalized to the mRNA level of each gene in normoxic embryos at 24 hpf with elongation factor 1-α (*eef1a1*) transcript levels as a control using the 2 − ΔΔCT method. For statistical analysis, student’s *t* test was used to compare normoxia and hypoxia groups.

### Availability of supporting data

The data sets supporting the results of this article are included within the article and its additional files. The RNAseq data is available at SRP062493.

## Additional files

Additional file 1: List of final 1270 genes for analysis (Ensembl gene ID listed). These genes were chosen based on known or likely roles in CNS connectivity development. (XLS 99 kb) Additional file 2: List of K-means cluster analysis of connectivity genes across development during normoxia. Relative log2 fold change compared to the developmental average is provided. (XLSX 171 kb) Additional file 3: List of K-means cluster analysis of connectivity genes across development during hypoxia. Relative log2 fold change compared to the developmental average is provided. (XLSX 197 kb) Additional file 4: Figure S1. GO analysis from Fig. 3 D and H, with GO terms displayed. Developmental profiles of connectivity genes (*n* = 1270), organized as K-means clusters with groups of genes showing similar expression pattern profiles across development. A) GO pathway analysis of K clusters in normoxia. B) GO pathway analysis of K clusters comparing hypoxia to normoxia. (JPG 1354 kb) Additional file 5: List of K means cluster analysis of connectivity genes across development during hypoxia, after averaging relative expression across development in both hypoxia and normoxia. Relative log2 fold change compared to the average is provided. (XLSX 257 kb) Additional file 6: Data sheets of qRT-PCR results for *atoh1a, arx, col5A3, htr3a,* and *zic2b*. First tab for each gene has raw data; second tab has analysis; third tab has statistical analyses. (XLSX 166 kb) Additional file 7: List of all genes analysis (Ensembl gene ID listed). Cluster assignment is shown; and relative counts in normoxia and hypoxia at different times. (XLSX 261 kb) Additional file 8: Data sheets of qRT-PCR results for *ryk*, *nrxn1a*, and *fezf2*. First tab for each gene has raw data; second tab has analysis; third tab has statistical analyses. (XLSX 109 kb) Additional file 9: Data sheets of connectivity genes; analyzed for unadjusted, adjusted for multiple comparisons, and FDR, across development and experimental conditions (comparing normoxia to hypoxia). (XLSX 3494 kb) Additional file 10: Figure S2. Larger file and font sizes showing gene names for the Protein-Protein Interactions Network. A) STRING analysis of most significant (adjusted *p* < 0.05) genes interactions, *n* = 57; color key for interaction type is shown to the right. B) STRING analysis with relaxed criteria (unadjusted *p* < 0.05), *n* = 244. (JPG 2997 kb)

## Abbreviations

ADHD: attention-deficit hyperactivity disorder
CNS: central nervous system
FACS: fluorescence-activated cell sorting
FDR: false discovery rate
FPKM: fragments per kilobase of transcript per million fragments mapped
GO: gene ontology
hif: hypoxia-inducible factor
hpf: hours post-fertilization
KEGG: Kyoto encyclopedia of genes and genomes
MRI: magnetic resonance imaging
PCA: principal component analysis
RT-PCR: reverse transcription polymerase chain reaction
TRAP: translating ribosomal affinity purification
