# Supplementary figures and images for: Temporal Dysynchrony in brain connectivity gene expression following hypoxia
Source: BMC Genomics. 2016 May 4;17:334. doi: 10.1186/s12864-016-2638-x (PMC4857255; doi:10.1186/s12864-016-2638-x)

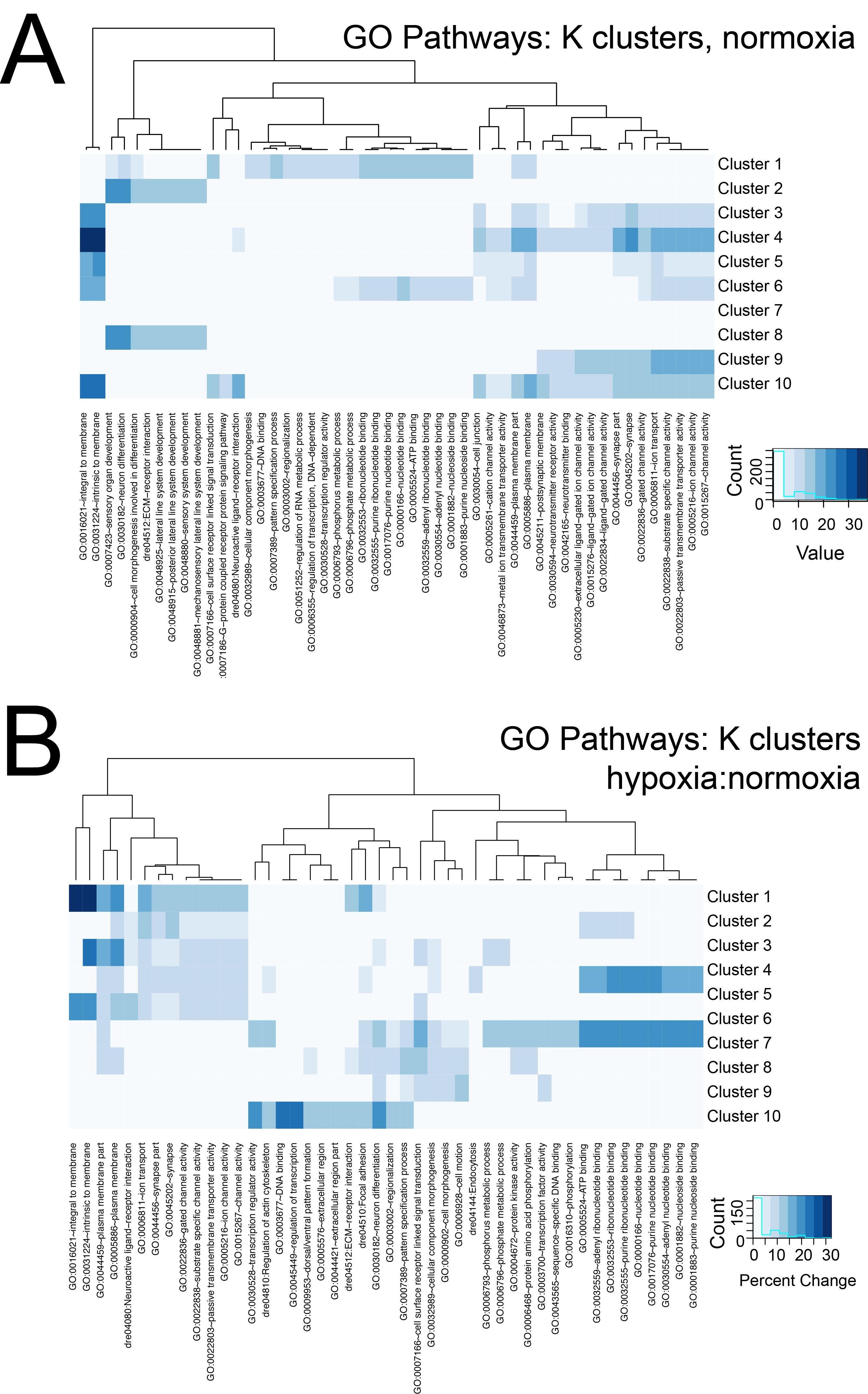

Supplement: Additional file 4: Figure S1. — GO analysis from Fig. 3 D and H, with GO terms displayed. Developmental profiles of connectivity genes (n = 1270), organized as K-means clusters with groups of genes showing similar expression pattern profiles across development. A) GO pathway analysis of K clusters in normoxia. B) GO pathway analysis of K clusters comparing hypoxia to normoxia. (JPG 1354 kb) [file 12864_2016_2638_MOESM4_ESM.jpg]

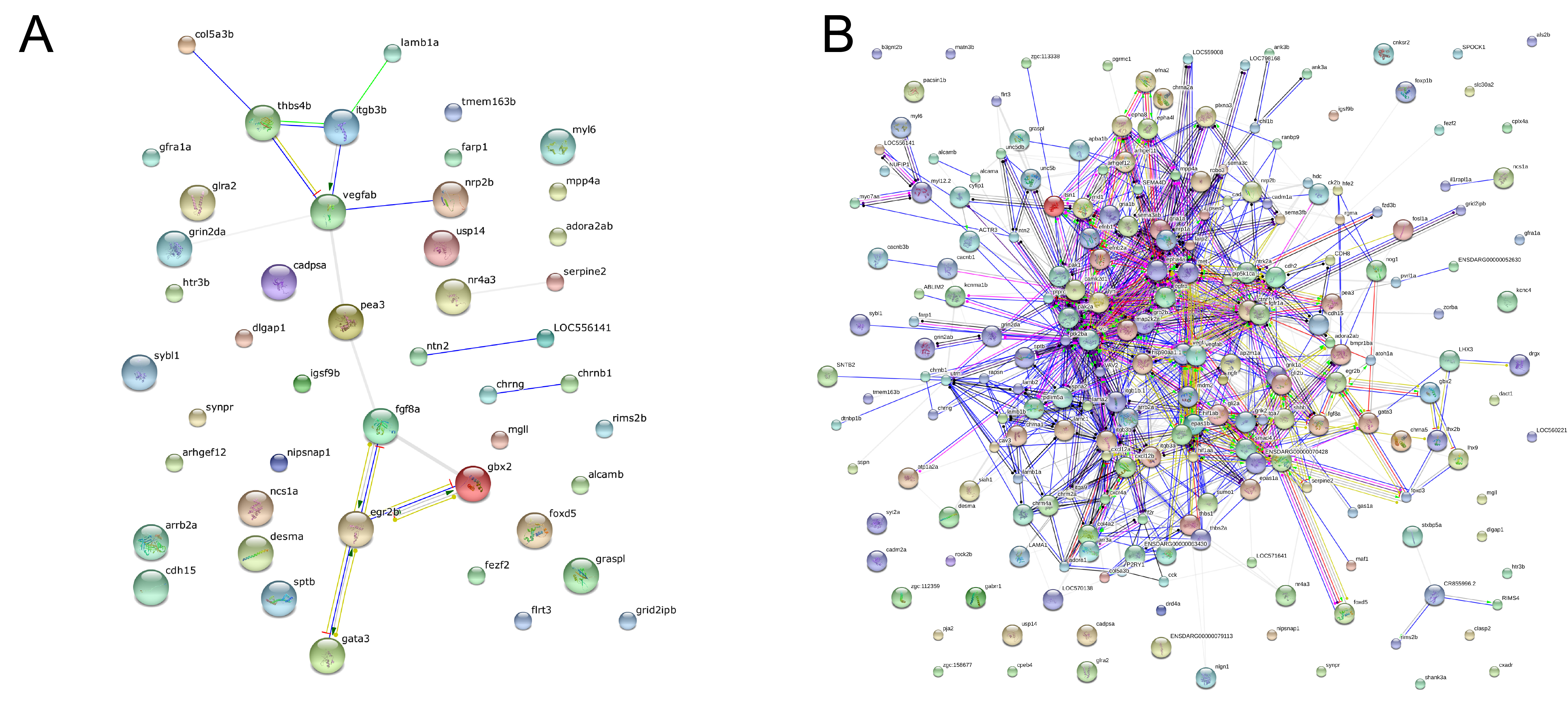

Supplement: Additional file 10: Figure S2. — Larger file and font sizes showing gene names for the Protein-Protein Interactions Network. A) STRING analysis of most significant (adjusted p < 0.05) genes interactions, n = 57; color key for interaction type is shown to the right. B) STRING analysis with relaxed criteria (unadjusted p < 0.05), n = 244. (JPG 2997 kb) [file 12864_2016_2638_MOESM10_ESM.jpg]
